# A115 EVALUATING THE CONTENT AND FACE VALIDITY OF LOW-COST GEL POLYPS FOR POLYPECTOMY SKILLS TRAINING IN NOVICE ENDOSCOPISTS

**DOI:** 10.1093/jcag/gwad061.115

**Published:** 2024-02-14

**Authors:** D Tham, A Zhao, N Gimpaya, K Khalaf, A Mokhtar, C Na, J Lisondra, M Scaffidi, R Khan, C Walsh, A Fecso, S Grover

**Affiliations:** St Michael's Hospital, Division of Gastroenterology, Toronto, ON, Canada; St Michael's Hospital, Division of Gastroenterology, Toronto, ON, Canada; St Michael's Hospital, Division of Gastroenterology, Toronto, ON, Canada; St Michael's Hospital, Division of Gastroenterology, Toronto, ON, Canada; St Michael's Hospital, Division of Gastroenterology, Toronto, ON, Canada; St Michael's Hospital, Division of Gastroenterology, Toronto, ON, Canada; St Michael's Hospital, Division of Gastroenterology, Toronto, ON, Canada; Medicine, Queen's University Faculty of Health Sciences, Kingston, ON, Canada; St Michael's Hospital, Division of Gastroenterology, Toronto, ON, Canada; Division of Gastroenterology, Hepatology, and Nutrition, SickKids Research and Learning Institutes, The Hospital for Sick Children, Toronto, ON, Canada; Minimally Invasive and Bariatric Surgery, Therapeutic Endoscopy, University Health Network, Toronto, ON, Canada; Scarborough Health Network Research Institute, Toronto, ON, Canada

## Abstract

**Background:**

Polypectomy is an essential endoscopic skill involving the removal of potentially premalignant lesions during colonoscopy. The *ad hoc* nature of polyp encounters is a significant barrier to polypectomy training, as this limits the ability to deliberately practice the skill. Effective and accessible polypectomy simulators may improve global implementation of polypectomy curricula.

**Aims:**

To determine the face and content validity of a novel low-cost gel polyp simulator for use in endoscopic training programs.

**Methods:**

A novel gel polyp simulator with multiple polyp morphologies based on the Paris classification was developed and used with a commercially available colonic phantom (Koken Lower GI Endoscopy Simulator Type II LM-107, Koken CO.LTD, Japan) in an endoscopy simulation-based training course (July, 2023). Feedback based on previous prototypes of the simulator were implemented into the simulator utilized in the present study. Novice (ampersand:003C25 procedures) and expert (ampersand:003E1000 procedures) endoscopists performed simulated procedures with the gel polyps. Using a five-point Likert scale (“strongly agree” to “strongly disagree”), a post-training survey based on previously published surveys used to determine the face and content validity of low-cost physical colonic simulators was administered. A section for additional comments was placed at the end of the survey.

**Results:**

The face and content validity survey was completed by 3 experts and 21 novices. The median score for all face validity questions was 5 (IQR 5-5) from experts and 5 (IQR 5-5) from novices. The median score for all content validity questions was 5 (IQR 5-4) from experts and 5 (IQR 5-5) from novices. In the additional comments section of the survey, one expert commented that future iterations of the gel polyps should be made thicker so that trainees could practice using endoscopic clips. The gel polyp simulator cost $0.61 CAD/polyp including the cost of materials and 3D printed molds.

**Conclusions:**

The gel polyp simulator appears to be a feasible modality for polypectomy training that is reproducible using accessible and low cost materials.

Face and Content Validity Survey (questions and median survey score)

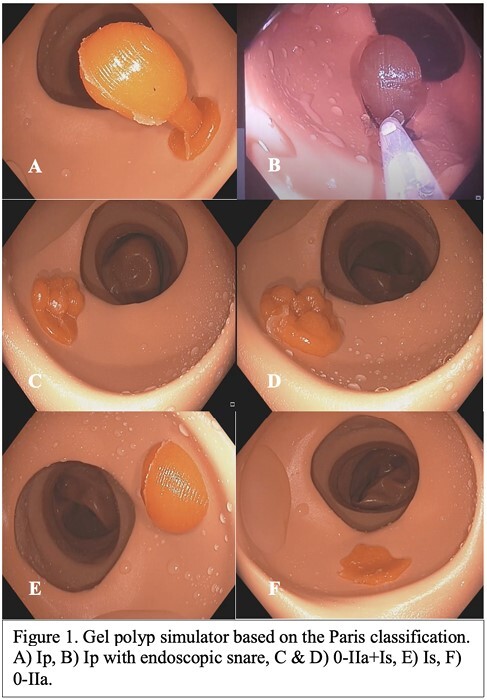

**Funding Agencies:**

None

**Table:** 

Face Validity Survey	Median Survey Score (Student/Teacher)
I believe that practicing with the gel polyp simulator would improve my specific skills.	5/5
I would like to continue exercising with the gel polyp simulation in further training.	5/5
I believe that training with the gel polyp simulator reduces the risks for patients.	5/5
I would recommend the gel polyp simulator.	5/5
Content Validity Survey	Median Survey Score (Student/Teacher)
The gel polyps were easy to handle (preparation, down time, complexity during the course).	5/4
Practicing with the gel polyps is advantageous during a training program.	5/5
The gel polyps can be easily integrated into a training program.	5/5
The gel polyps display enough realism for the practiced procedure.	5/4
Training with the gel polyps leads to a direct improvement of the trainee	5/5

